# Linking critical temperature with electron localization for cavity-enhanced superconductivity

**DOI:** 10.1038/s42005-026-02604-9

**Published:** 2026-04-13

**Authors:** Omid Nourmofidi, Hannes Hübener, E. K. U. Gross, Angel Rubio

**Affiliations:** 1 Max Planck Institute for the Structure and Dynamics of Matter and Center for Free-Electron Laser Science, Hamburg, Germany; 2 Quantum Dynamics Laboratory, Tsientang Institute of Advanced Study, Hangzhou, Zhejiang China; 3 Fritz Haber Center for Molecular Dynamics, Institute of Chemistry, The Hebrew University of Jerusalem, Jerusalem, Israel; 4 Initiative for Computational Catalysis, The Flatiron Institute, New York, NY USA

**Keywords:** Superconducting properties and materials, Nanocavities

## Abstract

Predicting superconducting properties from first principles—especially in non-equilibrium conditions—is computationally intensive. Here, we propose a more efficient approach by using the electron localization function (ELF) as a proxy for estimating the superconducting critical temperature *T*_C_. Through first-principles calculations, we investigate how coupling conventional superconductors to an optical cavity—without external driving—modifies their phonon properties and electron-phonon interactions via vacuum fluctuations alone. We focus on three representative materials: lead (Pb), niobium (Nb), and magnesium diboride (MgB_2_). Our methodology combines Density Functional Theory (DFT), Density Functional Perturbation Theory (DFPT), Quantum Electrodynamical Density Functional Theory (QEDFT), and Wannier-based electron-phonon coupling to solve the Eliashberg equations for *T*_C_. For the materials studied here, our results indicate that the ELF captures some trends in the superconducting behavior under light-matter coupling, suggesting it may serve as a low-cost descriptor to guide the screening or design of superconductors in equilibrium and cavity-modified regimes.

## Abstract (web summary)

Predicting how superconductors behave when coupled to light usually requires demanding first-principles calculations. Here, the authors show that the electron localization function can capture key trends in how vacuum light-matter coupling modifies the superconducting critical temperature, offering a potentially faster way to screen and design material.

## Introduction

Controlling material properties through light–matter interactions has become a powerful and versatile approach in condensed matter physics^1–10^. While strong laser fields enable dynamic manipulation of materials in out-of-equilibrium regimes^11–13^, an alternative equilibrium-based strategy harnesses the quantum fluctuations of confined electromagnetic fields within optical cavities^14–22^. These cavity-induced modifications can profoundly influence electronic, vibrational, and chemical properties, giving rise to emerging fields such as in polaritonic chemistry^23–26^ and cavity materials engineering^27,28^.

Superconductivity, defined by zero DC electrical resistance and perfect diamagnetism below a critical temperature *T*_C_, underpins a range of transformative technologies, from quantum computing^29^ and high-field magnets to magnetic resonance imaging (MRI), energy-efficient electronics, and lossless power transmission. The ongoing search for superconductors with higher *T*_C_ is central to enabling these applications at scale, particularly in settings where cryogenic cooling imposes major limitations.

While phonon-mediated superconductors are well described by Migdal-Eliashberg theory^30^ or superconducting DFT (SCDFT)^31,32^—both being a strong-coupling extension of BCS theory^33^—the situation is markedly different for high-*T*_C_ materials such as cuprates^34^ and some nickelates^35^. In these systems, the nature of the pairing mechanism remains unresolved, and no unified microscopic theory exists that can consistently explain or predict their superconducting behavior. Strong electronic correlations, competing orders, and non-trivial interactions with lattice and spin degrees of freedom make accurate theoretical modeling particularly challenging. The current high-*T*_C_-record holders reaching nearly room-temperature superconductivity are hydrogen-rich compounds under extreme pressure. These materials are largely understood within the standard phonon-mediated mechanism^36^.

A promising emerging approach for tuning superconducting properties involves cavity quantum electrodynamics, where materials are embedded in optical cavities that modify their electronic and vibrational structure through interactions with the quantum electromagnetic vacuum^37,38^. Importantly, this coupling can enhance or reshape superconducting properties without any external driving field, providing a fundamentally new equilibrium-based mechanism to engineer material behavior via light-matter interactions.

However, predicting the resulting Tc from first principles in such hybrid systems remains computationally demanding^39^. Even for conventional superconductors, simulating cavity-induced effects requires integrating advanced electronic structure methods—such as density functional theory (DFT)^40,41^ and density functional perturbation theory (DFPT)^42^—with quantum electrodynamical extensions (QEDFT)^43^ and solving the Eliashberg or, alternatively, the SCDFT equations. These challenges are compounded in non-equilibrium or strongly coupled regimes, where conventional approximations break down, and computational cost escalates rapidly^44^.

To accelerate progress, there is a critical need for physically meaningful, computationally tractable descriptors that correlate reliably with *T*_C_. Identifying such indicators would enable high-throughput screening and rational design of superconductors—both conventional and cavity-modified—paving the way for practical advances in quantum technologies, low-loss power systems, and next-generation hybrid optoelectronic devices.

To explore potential correlations between electron localization and *T*_C_, we calculate the electron localization function (ELF)^45,46^, a relatively inexpensive quantity that provides insight into electronic structure changes induced by cavity fields. In addition to ELF, we directly compute superconducting properties within standard Eliashberg theory. For the conventional electron-phonon superconductors considered here, we find that the ELF shows a qualitative relationship with *T*_C_, suggesting it may serve as a practical descriptor to help guide the exploration of superconducting behavior in cavity-modified systems influenced by vacuum fluctuations, as well as in light-driven or other non-equilibrium regimes, and providing a starting point for identifying trends and informing further studies.

In this work, we focus on three conventional phonon-meidated superconductors: Lead (Pb) with *T*_C_ ≈ 7.2 K, niobium (Nb) with *T*_C_ ≈ 9.25 K, and magnesium diboride (MgB_2_), which with a *T*_C_ of 39 K^47^ holds the highest known transition temperature among electron–phonon–mediated superconductors at ambient pressure^47^. We choose these materials to investigate the generality of the connection between ELF and *T*_C_ for electron-phonon superconductors with different electronic properties: Lead is a prototypical simple metal, niobium is a transition metal with localized *d* electrons, and MgB_2_ is a layered metallic material dominated by covalent bonding. Using first-principles quantum electrodynamical density functional theory (QEDFT)^38^, we investigate how placing these materials inside optical cavities modifies their electronic structures, phonon spectra, and ultimately their superconducting properties.

Our computational approach combines Density Functional Theory (DFT)^40,41^ and Density Functional Perturbation Theory (DFPT)^42^ to obtain electronic and vibrational properties. Electron–phonon interactions are then evaluated using the EPW code (Electron–Phonon coupling using Wannier functions)^39,48^, which leverages Maximally Localized Wannier Functions (MLWFs)^49^ for efficient interpolation of electron–phonon matrix elements. Inside the cavity, the electronic structure and phonons are computed within the QEDFT framework, while superconducting properties are extracted from standard Eliashberg calculations based on the cavity-modified input data. The ELF is computed within the (QE)DFT framework for both free-space and cavity-embedded materials.

With this study, we aim to deepen the understanding of how cavity quantum vacuum fluctuations impact superconductivity in representative materials and to assess the potential of electron localization as a predictive tool for superconducting transition temperatures. We combine DFT, DFPT, QEDFT, and Wannier-based electron–phonon coupling to evaluate cavity-induced modifications of phonons, electron–phonon interactions, and the resulting *T*_C_ in Pb, Nb, and MgB_2_. Our results show that vacuum fluctuations alone can influence superconducting-relevant properties and that the electron localization function captures key trends in the cavity-modified behavior, supporting its use as an efficient descriptor for screening and design.

## Results

### Cavity-modified phonon properties

**Fig. 1:**
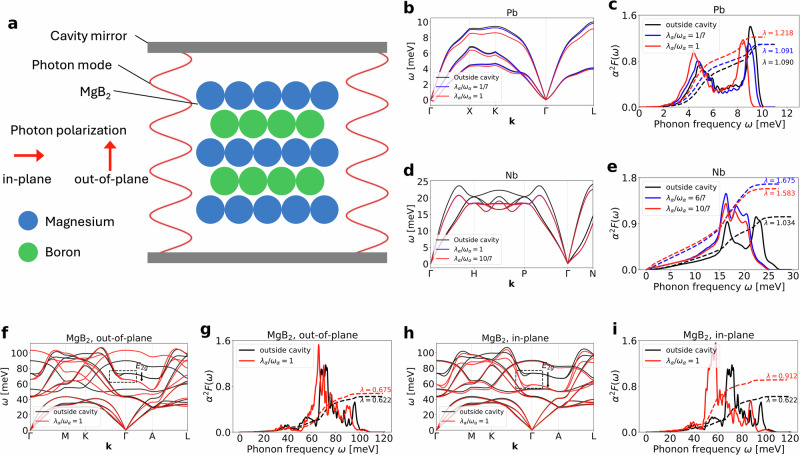
Changes to the phonon dispersion and isotropic Eliashberg spectral function inside a cavity. **a** Setup: electron–phonon superconductor (MgB_2_) inside an optical cavity, characterized by photon mode strength *λ*_*α*_, frequency *ω*_*α*_, and polarization **ϵ**_*α*_, following ref. ^38^. **b** Lead: phonon dispersion outside (black) and inside with three photon modes (*x*, *y*, *z*) at *λ*_*α*_/*ω*_*α*_ = 1/7 (blue) and 1 (red). Phonons soften inside the cavity. **c** Lead: Eliashberg spectral function *α*^2^*F*(*ω*) (solid) and *λ*(*ω*) (dashed) outside (black), inside at 1/7 (blue) and 1 (red). Cavity enhances spectral weight near 5 meV and suppresses it near 9 meV. **d** Niobium: phonon dispersion outside (black) and inside at *λ*_*α*_/*ω*_*α*_ = 1 (blue) and 10/7 (red). Phonons soften with increasing coupling. **e** Niobium: Eliashberg spectral function *α*^2^*F*(*ω*) (solid) and *λ*(*ω*) (dashed) outside (black), inside at 1 (blue) and 10/7 (red). Electron–phonon coupling is strongest for *λ*_*α*_/*ω*_*α*_ = 1. **f** MgB_2_: Phonon dispersion outside (black) and inside the cavity at *λ*_*α*_/*ω*_*α*_ = 1 for out-of-plane polarization and **g** Isotropic Eliashberg spectral function *α*^2^*F*(*ω*) (solid) and electron–phonon coupling *λ*(*ω*) (dashed) for the same case as (**b**). **h**, **i** Same as (**f**, **g**) but for in-plane polarization. Phonons are softened (including the *E*_2*g*_ mode driving Cooper pairing in MgB_2_), and electron–phonon coupling is enhanced, stronger for in-plane.

We consider those three materials both outside and inside an optical cavity. Magnesium diboride (MgB_2_) is a layered compound consisting of alternating layers of magnesium in a hexagonal lattice and boron atoms in a honeycomb lattice. The bonding is dominated by strong covalent *σ*-bonds between boron atoms, which form a two-dimensional network, and weaker *π*-bonds, which form a three-dimensional network^47^. The calculations for MgB_2_ are based on the methodology described in ref. ^38^. When MgB_2_ is placed inside an optical cavity, as illustrated in Fig. 1a, we study two configurations. In the first configuration, we use out-of-plane polarization with a single effective photon mode polarized perpendicular to the boron planes. In the second configuration, we use in-plane polarization with two effective photon modes, one polarized along the *x* direction and the other along the *y* direction, in order to preserve the in-plane symmetry of the lattice. For lead and niobium, we consider two cavity mirrors along perpendicular axes to accommodate three effective photon modes polarized along the *x*, *y*, and *z* directions. For the calculations inside the cavity, we use in all cases the DFT-relaxed lattice constants (see “Methods”).

We consider a dark cavity, i.e., we only take into account the vacuum fluctuations of the electromagnetic field. We note here that the ratio *λ*_*α*_/*ω*_*α*_, where *λ*_*α*_ is the mode strength of the *α*-th mode with ${\lambda }_{\alpha }=\sqrt{4\pi /{\Omega }_{\alpha }}$ with effective mode volume Ω_*α*_ and *ω*_*α*_ is its frequency, is treated as the only free parameter in our cavity setup^50^. This ratio includes all the properties necessary to describe the electron-photon interaction, since the electron-photon exchange potential used in our simulations depends only on this ratio (see “Methods”). At the current state of the art, there is no clear and systematic mapping between experimental setups—specifically cavity sizes and geometries—and the resulting values of the normalized light–matter coupling strength *λ*_*α*_/*ω*_*α*_. Nevertheless, the central objective in cavity-based light–matter engineering is to achieve extremely small mode volumes together with strongly spatially varying electromagnetic fields^51^. Conventional two-mirror cavities, such as Fabry–Pérot resonators, are unlikely to provide coupling strengths large enough to induce significant modifications of material properties^50^. In contrast, plasmonic nanocavities can support highly confined modes and large vacuum field amplitudes, suggesting that coupling strengths of at least *λ*_*α*_/*ω*_*α*_ ~ 0.1 should be experimentally accessible^52^.

We now investigate how the phonons are modified when the materials are placed inside the cavity. The phonon dispersions for lead and niobium, as well the two setups of MgB_2_, for selected coupling strengths *λ*_*α*_/*ω*_*α*_, are shown in Fig. 1b, d, f, h, respectively. As can be seen, the cavity has a similar effect on all three materials, namely a softening of the phonon modes, corresponding to lower-energy phonons for a given band and momentum. We also note the softening of the *E*_2*g*_ phonon mode in MgB_2_, which is the mode responsible for Cooper pairing^33^ and, consequently, superconductivity in MgB_2_^47^. For in-plane polarization, the softening is stronger than for out-of-plane polarization ref. ^38^, demonstrated that the effect of the cavity is to increase the electron density along the boron–boron bonds when placing MgB_2_ in a cavity, which can explain the softening of the phonon modes in this material due to stronger Coulomb screening, resulting in lower restoring forces and therefore lower-energy phonons.

In Fig. 1c, e, g, i, we show the computed isotropic Eliashberg spectral function *α*^2^*F*(*ω*), which describes the probability of an electron interacting (via emission or absorption) with a phonon of frequency *ω*, and the cumulative electron-phonon coupling strength *λ*(*ω*). We focus on the total electron-phonon coupling strength *λ*, which is given by *λ*(*ω*) evaluated at the highest phonon frequency (see Methods). An increase in the total electron-phonon coupling strength corresponds to an increase in the critical temperature in conventional phonon-mediated superconductors^53^. For the Eliashberg spectral function, we observe, in all cases, a shift towards lower energies, which is expected since the phonons themselves are softened inside the cavity.

For lead, Fig. 1c shows two pronounced peaks in the isotropic Eliashberg spectral function at approximately 4 and 9 meV. These peaks correspond to the relatively flat regions of the lower and upper phonon branches shown in Fig. 1b, indicating that these two branches play a dominant role in the superconducting pairing mechanism in lead. For a coupling parameter of *λ*_*α*_/*ω*_*α*_ = 0.1, the total electron–phonon coupling strength *λ* decreases slightly relative to the reference case. Inspection of the Eliashberg spectral function shows that this change arises from a redistribution of spectral weight: the low-energy peak is enhanced, whereas the high-energy peak is suppressed. The implications of this redistribution for the superconducting critical temperature will be discussed later. At stronger coupling, *λ*_*α*_/*ω*_*α*_ = 6/7, both peaks are enhanced compared to the weaker-coupling case, resulting in a larger total electron–phonon coupling constant.

For niobium, we consider the two coupling strengths *λ*_*α*_/*ω*_*α*_ = 6/7 and *λ*_*α*_/*ω*_*α*_ = 10/7. As shown in Fig. 1e, the isotropic Eliashberg spectral function and the total electron-phonon coupling strength reveal that the coupling is enhanced for *λ*_*α*_/*ω*_*α*_ = 6/7, but decreases again for the stronger coupling *λ*_*α*_/*ω*_*α*_ = 10/7.

For MgB_2_, we see in Fig. 1g for the out-of-plane polarization and in Fig. 1i for the in-plane polarization that the peak at approximately 70 meV, associated with the *E*_2*g*_ phonon mode, is enhanced inside the cavity for a coupling of *λ*_*α*_/*ω*_*α*_ = 1, particularly for in-plane polarization. We also find that the total electron-phonon coupling strength increases in both cases, with a stronger enhancement for in-plane polarization, suggesting a higher expected critical temperature in that configuration.

A qualitative explanation of why the in-plane polarization has a stronger influence than the out-of-plane polarization on the softening of the *E*_2*g*_ phonon mode, and consequently on the overall enhancement of the electron-phonon coupling, is as follows. The *E*_2*g*_ phonon mode is an in-plane mode within the boron plane. Therefore, to effectively modify electronic properties, such as their localization, in a way that influences the phonon modes, it is more efficient to apply a polarization within the plane rather than out of the plane.

Although MgB_2_ has the *E*_2*g*_ phonon mode, which is the dominant mode for superconductivity at around 70meV, we do not consider the cavity to be tuned such that its modes lie within the energy range of this phonon mode and couple resonantly to it. Instead, the cavity is tuned to the energy range of the electrons, thereby modifying electronic properties such as charge distribution and localization, which in turn influence the phonon modes. Thus, the changes in the phonon modes occur indirectly via modifications of the electronic properties that couple to the cavity. This mechanism is not only valid for MgB_2_, but also applies to the other materials considered.

### Cavity-modified electron localization function

**Fig. 2:**
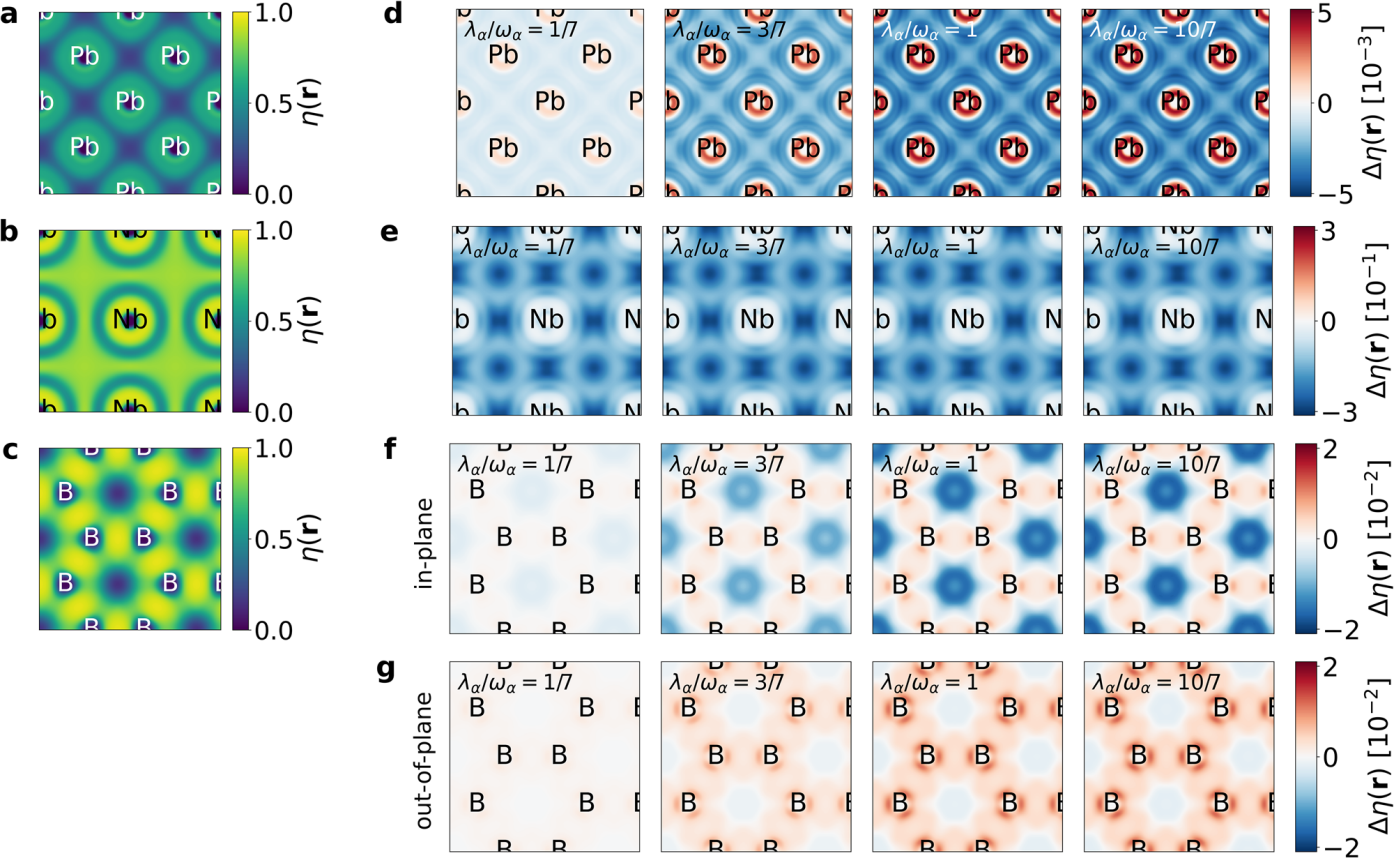
Change of the Electron Localization Function (ELF) inside an optical cavity. **a** Lead: ELF *η*(**r**) outside the cavity, mostly around 0.5, typical of a metallic element. **b** Niobium: ELF outside the cavity, due to strong localization in *d* bonds. **c** MgB_2_: ELF in the boron layer outside the cavity. Electrons are delocalized near boron ions and hexagon centers, but strongly localized in the bonds, indicating covalent bonding. **d** Lead: Change in ELF Δ*η*(**r**) inside the cavity for *λ*_*α*_/*ω*_*α*_ = 1/7, 3/7, 1, 10/7 with the mode strength*λ*_*α*_ and frequency *ω*_*α*_ of the *α*-th photon mode compared to the case outside the cavity (**a**). Electrons localize more strongly near ions and delocalize in the interstitial regions. **e** Niobium: Change in ELF inside the cavity for *λ*_*α*_/*ω*_*α*_ = 1/7, 3/7, 1, 10/7. Electrons delocalize between ions, but the overall change with coupling strength is small. **f** MgB_2_: Change in ELF inside the cavity for out-of-plane polarization at coupling strengths *λ*_*α*_/*ω*_*α*_ = 1/7, 3/7, 1, 10/7 (Δ*η* = *η*_cavity_ − *η*). Electrons localize more strongly around boron atoms and bonds. **g** MgB_2_: Same as (**f**) but for in-plane polarization. Localization is again enhanced, but weaker than in the out-of-plane case. Delocalization of electrons in the middle of the hexagons is stronger in the in-plane case, however.

We have seen how the cavity influences the phonon dispersion, and now we turn to the electron localization function (ELF) *η*(**r**). Since we aim to establish the ELF as a proxy quantity, it is necessary to analyze how it changes inside the cavity, in analogy to the phonons. To this end, we examine the ELF in a two-dimensional plane within each material. The ELF measures the degree of localization of electrons in atoms, molecules, and solids at a given position **r** (see “Methods”)^45,46^. Its range extends from 0 (complete delocalization) to 1 (complete localization). It is important to emphasize that the ELF is not equivalent to the electron charge density. Therefore, the changes shown here do not represent a redistribution of charge, but rather variations in electron localization. The electron localization of lead, niobium, and MgB_2_ are shown in Fig. 2a–c. For lead and niobium, we consider the (001) plane. For MgB_2_, we consider the boron plane, since the *E*_2*g*_ phonon mode is present only within the boron layer, and thus our analysis focuses on that layer. It is important to note that the electrons considered in the ELF are only those treated explicitly in the DFT calculation, which uses pseudopotentials to account for core electrons. Hence, the contribution of the localized core electrons are not considered in this description.

For lead, the ELF has a value of around 0.5 in most regions. This is expected for a metallic element such as lead, and in a homogeneous free-electron gas^54,55^ the ELF takes a value of approximately 0.5. In metals, electrons can often be approximated by a homogeneous free-electron gas, consistent with this observation. For niobium, the electrons appear highly localized. This can be explained by the fact that bonding in niobium is dominated by *d*-orbitals, which are generally more localized than *s* or *p* orbitals. Furthermore, as niobium is a transition metal, the homogeneous free-electron gas approximation does not apply well. For MgB_2_, we observe low localization at the boron atoms and in the centers of the hexagons, but very high localization between the boron atoms. This is characteristic of covalent bonding, and the strong localization between the boron atoms confirms the presence of strong covalent *σ* bonds.

We now investigate how the ELF changes when the materials are placed inside an optical cavity. In Fig. 2d–g, we show the change in the electron localization for lead and niobium, as well as for MgB_2_ with out-of-plane polarization and in-plane polarization, for coupling strengths *λ*_*α*_/*ω*_*α*_ = 1/7, 3/7, 1, 10/7. Specifically, we show the difference between the ELF outside the cavity, *η*(**r**), and the ELF inside the cavity, *η*_cavity_(**r**), defined as Δ*η*(**r**) = *η*_cavity_(**r**) − *η*(**r**). A negative value of Δ*η*(**r**) indicates decreased localization at a given point, while a positive value indicates increased localization.

For lead, we show the change in electron localization in one layer in Fig. 2d. The cavity increases localization at the lead atoms but decreases it between the atoms, with negligible additional changes beyond *λ*_*α*_/*ω*_*α*_ = 1. The increased localization at the ions can be explained by the fact that, according to the Pauli–Fierz Hamiltonian, one consequence of light-matter interaction is an additional term in the Hamiltonian that counteracts the kinetic term, effectively increasing the electron mass^43^. As a result, the electrons behave more classically, and in the classical limit, they tend to localize closer to the positively charged ions.

The change in ELF for niobium inside the cavity is shown in Fig. 2g, where we find increased delocalization between the atoms but no increased localization at the ions. As for MgB_2_, the effect does not grow further for stronger couplings.

For MgB_2_, the electrons become more localized around the boron atoms and along the boron-boron bonds, with a stronger effect for out-of-plane polarization (Fig. 2f) than for in-plane polarization (Fig. 2g). The localization increases with coupling strength, but changes become small beyond *λ*_*α*_/*ω*_*α*_ = 1/7. We also observe increased delocalization in the centers of the hexagons, an effect that is more pronounced for in-plane polarization. Here too, the delocalization saturates for coupling strengths above *λ*_*α*_/*ω*_*α*_ = 1.

### Cavity-modified critical temperature and connection to ELF

**Fig. 3:**
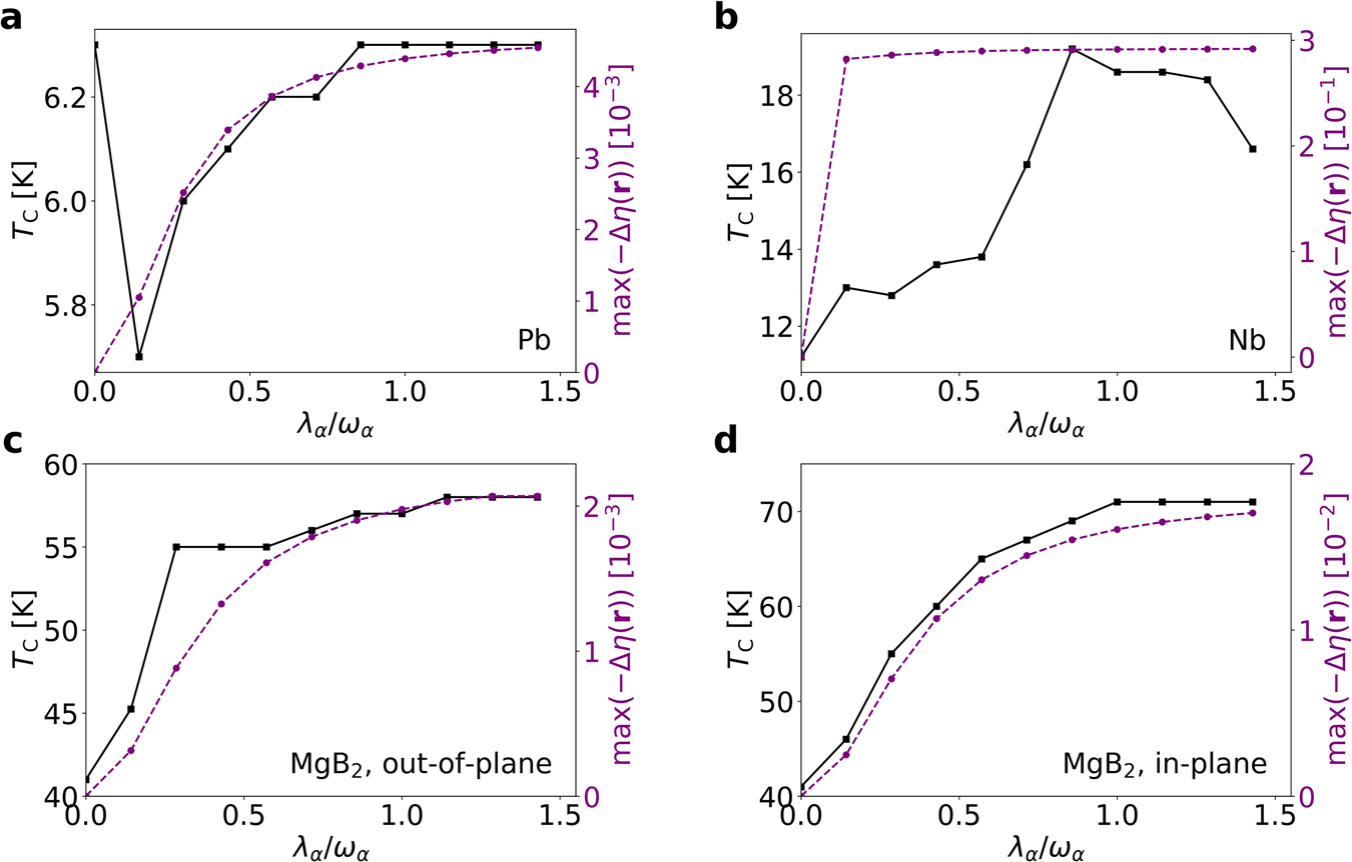
Trends for critical temperature *T*_C_ and Electron Localization Function (ELF) inside the cavity. Critical temperature *T*_C_ and maximum electron delocalization $\max (-\Delta \eta ({{{\bf{r}}}}))$ as a function of coupling strength *λ*_*α*_/*ω*_*α*_, with mode strength*λ*_*α*_ and frequency *ω*_*α*_ of the *α*-th photon mode. **a** Lead: *T*_C_ decreases initially but recovers at larger coupling. The Electron Localization Function (ELF) captures this behavior except for the initial drop. **b** Niobium: *T*_C_ increases significantly up to *λ*_*α*_/*ω*_*α*_ ≈ 1, then saturates. The ELF does not reproduce this trend. **c** MgB_2_ with out-of-plane polarization: *T*_C_ (black) and ELF delocalization (purple). Both increase with coupling and saturate for *λ*_*α*_/*ω*_*α*_ ≳ 1. **d** MgB_2_ with in-plane polarization: similar trend as in (**c**), with slightly stronger enhancement.

To establish the ELF as a reliable proxy for superconductivity, we now analyze how the critical temperature changes inside the cavity and draw correlations with the previously observed variations in the ELF. To calculate *T*_C_, the superconducting gap is determined from the Eliashberg equations^30^ at different temperatures. The highest temperature at which a non-trivial gap exists is defined as the critical temperature. The change of *T*_C_ as a function of the coupling strength *λ*_*α*_/*ω*_*α*_ for the three materials is shown in Fig. 3. In our discussion, we start with MgB_2_ to stress a point, which will be relevant for the other materials.

As seen in Fig. 3c, d, the critical temperature increases for MgB_2_ inside the cavity, reaching up to 58 K for out-of-plane polarization and 71 K for in-plane polarization. As expected from the isotropic Eliashberg spectral function *α*^2^*F*(*ω*) and the total electron-phonon coupling strength *λ* shown in Fig. 1g, i for out-of-plane and in-plane polarization, respectively, the increase in *T*_C_ is due to enhanced electron-phonon coupling, which is stronger for the in-plane case. The critical temperature saturates for *λ*_*α*_/*ω*_*α*_ ≳ 1 in both polarizations, consistent with the observation that the ELF does not change significantly beyond this coupling strength (see Fig. 2f, g.)

Before discussing the other two materials, we note one important aspect. Previous first-principles calculations^38^ show that the electron density in MgB_2_ increases along the boron-boron bonds when placed inside an optical cavity. However, for the out-of-plane polarization—which yields a lower *T*_C_—the gain in bond charge density is actually higher than for in-plane polarization. Therefore, electron density alone cannot be used to predict changes in *T*_C_. Our ELF calculations confirm that localization around boron atoms and bonds is stronger for out-of-plane polarization. However, delocalized electrons are more effective at screening Coulomb interactions, as they can respond more readily to the electric fields of the ions^56^. We suggest that enhanced Coulomb screening reduces the restoring forces of phonons, leading to phonon softening and consequently stronger electron-phonon coupling, which increases *T*_C_.

Examining the ELF changes inside the cavity (Fig. 2f, g), we observe that electron delocalization in the hexagon centers is larger for in-plane polarization, which may explain the higher *T*_C_. This suggests that the ELF, particularly the degree of electron delocalization, can serve as an indicator of the critical temperature by reflecting phonon softening and electron-phonon coupling strength.

To test whether ELF delocalization can serve as a proxy for *T*_C_, we also plot, alongside *T*_C_, the maximum decrease of the ELF in the boron layer, $\max (-\Delta \eta ({{{\bf{r}}}}))$, in Fig. 3. For MgB_2_, the ELF correlates well with *T*_C_, capturing both the overall increase and the small enhancement beyond *λ*_*α*_/*ω*_*α*_ = 1.

The critical temperature as a function of coupling strength for lead is shown in Fig. 3a. The critical temperature outside the cavity is approximately 6.3 K. Interestingly, for a small coupling strength of *λ*_*α*_/*ω*_*α*_ = 1/7, the critical temperature drops to 5.6 K. As the coupling increases further, the critical temperature gradually recovers, and at *λ*_*α*_/*ω*_*α*_ = 6/7 it reaches the same value as in the cavity-free case. Beyond this point, however, no significant enhancement is observed, indicating that the optical cavity does not improve the superconducting properties of lead. When considering the Eliashberg spectral function (ELF), we find that—apart from the initial drop—the ELF reproduces the trend of the critical temperature. For lead, the phonon density of states exhibits two peaks of comparable height^57–59^, which correspond to the features observed in the Eliashberg spectral function. Since in the Eliashberg spectral function the peak at around 9 meV is higher, these phonons couple more strongly to the electrons, and it is precisely these modes that are initially suppressed by the cavity. The lower-energy phonons are enhanced by the cavity, but they couple only weakly to the electrons.

In Fig. 3b, we also show the change of the critical temperature for different coupling strengths of niobium. It can be seen that the critical temperature increases significantly when niobium is placed inside the cavity, reaching up to *T*_C_ = 19.2 K for a coupling strength of approximately *λ*_*α*_/*ω*_*α*_ = 6/7. Beyond this point, the critical temperature remains nearly constant and even slightly decreases for very strong coupling. A similar behavior was also observed for MgB_2_, while in the case of lead, we found a saturation of the critical temperature at couplings beyond *λ*_*α*_/*ω*_*α*_ = 1. Even though the change in the ELF does not exactly follow the curve of the critical temperature for niobium, a consistent uptrend toward a coupling of *λ*_*α*_/*ω*_*α*_ = 1 can still be observed. In agreement with our conclusions from the previous examples, the ELF predicts increased delocalization of electrons between the atoms, which is accompanied by an increase in the critical temperature. This strengthens our argument that enhanced electron delocalization in the interatomic regions may be linked to higher critical temperatures.

## Discussion

In this work, we have investigated how the superconducting properties of selected materials change inside an optical cavity that is not externally driven by a laser but instead utilizes only the vacuum fluctuations of the electromagnetic field. In addition, we analyzed the electron localization function (ELF) to explore potential correlations with superconducting behavior.

For MgB_2_, we observed significant changes in the superconducting properties. The ELF shows that electrons tend to delocalize between boron atoms in the middle of the hexagons, which influences the phonon behavior. A qualitative correlation between localization and the critical temperature suggests the ELF may serve as an indicator for superconducting properties.

For lead, the changes in superconducting properties are less pronounced. The phonon spectrum is altered, with one peak in the Eliashberg spectral function increasing while the other decreases. This mixed behavior impacts the critical temperature, which slightly decreases at weak coupling but recovers at stronger coupling. ELF analysis again shows delocalization between the atoms, but no clear correlation with *T*_C_ is established in this case.

For niobium, we observed a non-monotonic behavior of the critical temperature. Initially, it increases, but at very strong coupling, it decreases again. This trend is supported by the Eliashberg spectral function. Here, we again observe that increased delocalization between atoms is associated with an enhancement of the critical temperature. Even though the ELF does not mirror the shape of the critical temperature curve, it still exhibits a well-defined uptrend up to strong couplings. The mismatch of the two curves may arise from the presence of complex bonding environments.

It is clear that the cavity-induced changes in the localization of electrons not only modify the phonons and the electron-phonon coupling, but also the screening among the electrons and hence μ* as well as *T*_C_. This effect, not considered here, is expected to be less important than the changes in the phonon-related properties^60^. We also note that, in the case of a Fabry–Pérot cavity, the photon modes are transverse and couple only to the electrons, since we assume the cavity modes to lie within the energy range of electronic excitations. Because these modes are transverse, they do not affect the longitudinal Coulomb interaction between electrons. Consequently, the Coulomb pseudopotential μ* is not expected to change significantly, and we assume it to remain constant when tuning the cavity coupling for all three materials^61^. In contrast, under the Born–Oppenheimer approximation, the cavity does not couple to the nuclei, as the cavity modes are assumed to lie outside the energy range of nuclear excitations. Therefore, the previous argument does not hold for the nuclei: the Coulomb interaction between them can be significantly modified due to the change of environment, thereby influencing the phonon modes, as discussed above.

These results suggest that it may be possible to define a quantity derived from the ELF that serves as a useful descriptor for classifying materials and for establishing qualitative connections to superconducting properties, such as the critical temperature. While further validation is clearly required, this approach can help narrow the search space for new superconducting materials. In addition, ELF-based descriptors may offer guidance for experiments in which materials are driven out of equilibrium, for example, by laser excitation, to explore potential enhancements of superconductivity. Given that direct calculations of superconducting properties under non-equilibrium conditions are computationally demanding and often difficult to converge, ELF-based approaches^62^ could provide a practical and complementary means to inform and guide such studies.

As an outlook, further work will be needed to extend this analysis to a broader set of materials and to different classes of superconductors, including systems beyond conventional electron–phonon-mediated superconductivity. Such studies would help clarify the range of applicability of ELF-based descriptors and assess whether the observed connection between the average ELF and the critical temperature remains robust across different superconducting mechanisms.

## Methods

### Quantum-electrodynamical density functional theory (QEDFT)

The interaction of non-relativistic electrons in matter with light is described by the Pauli-Fierz (PF) Hamiltonian^43^. In the velocity gauge and long wavelength approximation^50^, is given by

1 $${\widehat{H}}_{{{{\rm{PF}}}}} = 	 \frac{1}{2}{\sum }_{l=1}^{{N}_{e}}{\left(-i{\nabla }_{l}+\frac{1}{c}\widehat{{{{\bf{A}}}}}\right)}^{2}+\frac{1}{2}{\sum }_{l\ne k}^{{N}_{e}}w({{{{\bf{r}}}}}_{l},{{{{\bf{r}}}}}_{k})\\ 	 +{\sum }_{l=1}^{{N}_{e}}{v}_{{{{\rm{ext}}}}}({{{{\bf{r}}}}}_{l})+{\sum }_{\alpha =1}^{{M}_{p}}{\omega }_{\alpha }\left({\widehat{a}}_{\alpha }^{{{\dagger}} }{\widehat{a}}_{\alpha }+\frac{1}{2}\right),$$

where we use Hartree atomic units. *w*(**r**_*l*_, **r**_*k*_) is the Coulomb interaction, *v*_ext_(**r**_*l*_) is the external potential by the nuclei, and **r**_*l*_ is the position of the *l*th electron, and *ω*_*α*_ is the photon frequency and ${\widehat{a}}_{\alpha }^{{{\dagger}} }$ (${\widehat{a}}_{\alpha }$) are the creation (annihilation) operators of the *α*-th effective photon mode. The vector potential in the long wavelength approximation is given by $\widehat{{{{\bf{A}}}}}=c{\sum }_{\alpha =1}^{{M}_{p}}{\lambda }_{\alpha }{{{{\boldsymbol{\varepsilon }}}}}_{\alpha }\left({\widehat{a}}_{\alpha }^{{{\dagger}} }+{\widehat{a}}_{\alpha }\right)/\sqrt{2{\omega }_{\alpha }}$. Here, *c* is the speed of light and ***ε***_*α*_ is the polarization vector of the *α*-th effective photon mode. The effective mode strength goes with $\propto 1/\sqrt{{\Omega }_{\alpha }}$, where Ω_*α*_ is the effective mode volume of the *α*-th effective photon mode.

Multiplying out the first term of the Pauli Fierz Hamiltonian (Eq. (1)) gives one term proportional to ${\widehat{{{{\bf{A}}}}}}^{2}$, which can be absorbed in the bare photons, which yields to a transformation from bare photons into dressed photons, which are denoted by the frequency ${\widetilde{\omega }}_{\alpha }$ and dressed photon creation and annihilation operators ${\widehat{a}}_{\alpha }^{{{\dagger}} }$ and ${\widehat{a}}_{\alpha }$, respectively^63,64^. The dressed photon frequency is given by ${\widetilde{\omega }}_{\alpha }^{2}=\sqrt{{\omega }_{\alpha }^{2}+{N}_{e}^{2}{\lambda }_{\alpha }^{2}}$. The dressed mode strength ${\widetilde{\lambda }}_{\alpha }$ and dressed polarization vector $\widetilde{{{{\boldsymbol{\epsilon }}}}}$ can be obtained via a unitary transformation^63,64^.

With the paramagnetic current operator ${{{{\bf{J}}}}}_{p}={\sum }_{l=1}^{{N}_{e}}(-i{\nabla }_{l})$ and the vector potential $\widehat{\widetilde{{{{\bf{A}}}}}}=c{\sum }_{\alpha =1}^{{M}_{p}}{\widetilde{\lambda }}_{\alpha }{\widetilde{{{{\boldsymbol{\varepsilon }}}}}}_{\alpha }\frac{1}{\sqrt{2{\widetilde{\omega }}_{\alpha }}}\left({\widehat{\widetilde{a}}}_{\alpha }^{{{\dagger}} }+{\widehat{\widetilde{a}}}_{\alpha }\right)$ we get for the Pauli-Fierz Hamiltonian the following expression:

$${\widehat{H}}_{{{{\rm{PF}}}}} = 	 -\frac{1}{2}{\sum }_{l=1}^{{N}_{e}}{\nabla }_{l}^{2}+\frac{1}{2}{\sum }_{l\ne k}^{{N}_{e}}w({{{{\bf{r}}}}}_{l},{{{{\bf{r}}}}}_{k})+{\sum }_{l=1}^{{N}_{e}}{v}_{{{{\rm{ext}}}}}({{{{\bf{r}}}}}_{l})\\ 	 +\frac{1}{c}\widehat{\widetilde{{{{\bf{A}}}}}}\cdot {\widehat{{{{\bf{J}}}}}}_{p}+{\sum }_{\alpha =1}^{{M}_{p}}\widetilde{{\omega }}_{\alpha }\left({\widehat{\widetilde{a}}}_{\alpha }^{{{\dagger}} }{\widehat{\widetilde{a}}}_{\alpha }+\frac{1}{2}\right),$$

The next step is to incorporate the effect of quantum fluctuation to the electronic system. For that, one considers the equations of motion of the dressed photonic annihilation and creation operators and, using an adiabatic approximation, yields a Breit type approximation for the term photonic term ${\widetilde{\omega }}_{\alpha }{\widehat{\widetilde{a}}}_{\alpha }^{{{\dagger}} }{\widehat{\widetilde{a}}}_{\alpha }\approx \frac{{\widetilde{\lambda }}_{\alpha }^{2}}{2{\widetilde{\omega }}_{\alpha }^{2}}{({\widetilde{\epsilon }}_{\alpha }\cdot {\widehat{J}}_{p})}^{2}$^64^. Using $\widehat{{{{\bf{A}}}}}={\sum }_{\alpha }{\widehat{A}}_{\alpha }{\widetilde{{{{\boldsymbol{\varepsilon }}}}}}_{\alpha }$ and the mode resolved Maxwell equation ${\widehat{A}}_{\alpha }=-c\frac{{\widetilde{\lambda }}_{\alpha }^{2}}{{\widetilde{\omega }}_{\alpha }^{2}}{\widetilde{{{{\boldsymbol{\varepsilon }}}}}}_{\alpha }\cdot {\widehat{{{{\bf{J}}}}}}_{P}$ we obtain a new Hamiltonian ${\widehat{H}}_{B}$, which captures photon field fluctuations through current-current fluctuations^38^:

2 $${\widehat{H}}_{B} = 	 -\frac{1}{2}{\sum }_{l=1}^{{N}_{e}}{\nabla }_{l}^{2}+\frac{1}{2}{\sum }_{l\ne k}^{{N}_{e}}w({{{{\bf{r}}}}}_{l},{{{{\bf{r}}}}}_{k})+{\sum }_{l=1}^{{N}_{e}}{v}_{{{{\rm{ext}}}}}({{{{\bf{r}}}}}_{l})\\ 	 +{\sum }_{\alpha =1}^{{M}_{p}}{\widetilde{\omega }}_{\alpha }-{\sum }_{\alpha =1}^{{M}_{p}}\frac{{\widetilde{\lambda }}_{\alpha }^{2}}{2{\widetilde{\omega }}_{\alpha }^{2}}{\left({\widetilde{{{{\boldsymbol{\epsilon }}}}}}_{\alpha }\cdot {\widehat{{{{\bf{J}}}}}}_{p}\right)}^{2},$$

where the last term in Eq. (2) contains the current-current correlation operator ${\left({\widetilde{{{{\boldsymbol{\epsilon }}}}}}_{\alpha }\cdot {\widehat{{{{\bf{J}}}}}}_{p}\right)}^{2}$, which counteracts the kinetic energy term, effectively enhancing the electron mass along the polarization direction^38^. By increasing the coupling strength, the effective mass of the electrons is increased, which yields to a more classical behavior as they tend to accumulate in regions of minimum external potential^63,65^.

For our calculations, we use the framework of standard DFT^40,41^ and to the standard Kohn-Sham Hamiltonian potential, which interoperates the electron–photon potential. Since our ansatz is photon-free, we do not need to consider the pure photons in the Kohn-Sham system^63,64^. So, the Kohn–Sham (KS) Hamiltonian is given by

3 $${\widehat{H}}_{{{{\rm{KS}}}}}=-\frac{1}{2}{\nabla }^{2}+{v}_{{{{\rm{KS}}}}}({{{\bf{r}}}})=-\frac{1}{2}{\nabla }^{2}+{v}_{{{{\rm{ext}}}}}({{{\bf{r}}}})+{v}_{{{{\rm{Hxc}}}}}({{{\bf{r}}}})+{v}_{{{{\rm{pxc}}}}}({{{\bf{r}}}}),$$

where *v*_KS_(**r**) is the total KS potential, consisting of the electron–electron interaction potential *v*_Hxc_(**r**), which includes the Hartree and exchange-correlation (xc) part and the electron-photon exchange potential *v*_pxc_(**r**). For the electron-electron interaction, standard DFT functionals are used^66^.

The photon exchange-correlation potential within the Local density approximation used in this work is given by the solution of the Poisson equation:

4 $${\nabla }^{2}{v}_{{{{\rm{pxLDA}}}}}({{{\bf{r}}}})=-{\sum }_{\alpha =1}^{{M}_{p}}\frac{2{\pi }^{2}{\widetilde{\lambda }}_{\alpha }^{2}}{{\widetilde{\omega }}_{\alpha }^{2}}{\left({\widetilde{{{{\boldsymbol{\epsilon }}}}}}_{\alpha }\cdot \nabla \right)}^{2}{\left(\frac{3\rho ({{{\bf{r}}}})}{8\pi }\right)}^{2/3},$$

The derivation of this can be found in refs. ^63,64^. Here *ρ*(**r**) is the electron density. We note that the photon exchange-correlation potential only depends on the ratio between the coupling strength ${\widetilde{\lambda }}_{\alpha }$ and the photon frequency ${\widetilde{\omega }}_{\alpha }$. Therefore, this ratio ${\widetilde{\lambda }}_{\alpha }/{\widetilde{\omega }}_{\alpha }$ serves in our work as the tuning parameter for the setup, instead of considering explicit quantities like the mode volume, photon frequency, cavity material, finesse, etc. For our calculation, the electron-photon exchange potential was implemented into to the Quantum Espresso (QE) PW package^67,68^.

To compute phonon properties, we use the ground-state solutions from QEDFT and apply density functional perturbation theory (DFPT)^42^. In standard DFPT, one needs to have an expression for the linear response of the external, Hartree, and exchange-correlation potential to the change of the electron density. By adding the electron-photon exchange potential to the standard Kohn-Sham potential (Eq. (3)), we need an expression for the change of the photon exchange potential as well. The response of the photon exchange-correlation potential to variations in electron density is given by:

5 $$\frac{\delta {v}_{{{{\rm{pxLDA}}}}}({{{\bf{r}}}})}{\delta \rho ({{{{\bf{r}}}}}^{{\prime} })} = 	 {\left(\frac{3}{8\pi }\right)}^{2/3}{\sum }_{\alpha =1}^{{M}_{p}}\frac{\pi {\widetilde{\lambda }}_{\alpha }^{2}}{3{\widetilde{\omega }}_{\alpha }^{2}}{\left[\rho ({{{{\bf{r}}}}}^{{\prime} })\right]}^{-1/3}\\ 	 \times \left\{{\left({\widetilde{{{{\boldsymbol{\epsilon }}}}}}_{\alpha }\cdot {\nabla }^{{\prime} }\right)}^{2}\frac{1}{| {{{\bf{r}}}}-{{{{\bf{r}}}}}^{{\prime} }| }\right\}.$$

This response term in terms of the plane-wave basis set was added to the QE PHONON package^67,68^ for this work.

### Eliashberg theory

For the computation of superconducting properties, we solve the anisotropic Migdal-Eliashberg equations^30^. Solving these equations requires, in general, the information of the electrons, phonons, and electron–phonon interaction, which are obtained from previous PW and PHONON calculations. Through the framework of QEDFT, the electron-photon interaction is incorporated in the electrons, phonons, and electron–phonon interaction. The anisotropic Eliashberg equations^69^ are given by:

6 $$Z(n{{{\bf{k}}}},i{\omega }_{l}) = 	 1+\frac{\pi T}{{N}_{F}{\omega }_{l}}{\sum }_{m{{{{\bf{k}}}}}^{{\prime} },{l}^{{\prime} }}\frac{{\omega }_{{l}^{{\prime} }}}{\sqrt{{\omega }_{{l}^{{\prime} }}^{2}+{\Delta }^{2}(m{{{{\bf{k}}}}}^{{\prime} },i{\omega }_{{l}^{{\prime} }})}}\\ 	 \times \lambda (n{{{\bf{k}}}},m{{{{\bf{k}}}}}^{{\prime} },l-{l}^{{\prime} })\,\delta ({\epsilon }_{n{{{\bf{k}}}}}),$$

7 $$Z(n{{{\bf{k}}}},i{\omega }_{l})\Delta (n{{{\bf{k}}}},i{\omega }_{l}) = 	 \frac{\pi T}{{N}_{F}{\omega }_{l}}{\sum }_{m{{{{\bf{k}}}}}^{{\prime} },{l}^{{\prime} }}\frac{\Delta (m{{{{\bf{k}}}}}^{{\prime} },i{\omega }_{{l}^{{\prime} }})}{\sqrt{{\omega }_{{l}^{{\prime} }}^{2}+{\Delta }^{2}(m{{{{\bf{k}}}}}^{{\prime} },i{\omega }_{{l}^{{\prime} }})}}\\ 	 \times \left[\lambda (n{{{\bf{k}}}},m{{{{\bf{k}}}}}^{{\prime} },l-{l}^{{\prime} })-{N}_{F}{V}_{n{{{\bf{k}}}},m{{{{\bf{k}}}}}^{{\prime} }}\right]\delta ({\epsilon }_{n{{{\bf{k}}}}}),$$

where Δ(*nk*, *iω*_*l*_) is the momentum-resolved (**k**-resolved) superconducting gap and *Z*(*nk*, *iω*_*l*_) is the mass renormalization function. *T* denotes the temperature and *ω*_*l*_ = (2*l* + 1)*πT* are the Matsubara frequency with integer values for *l*. For the calculation of the Coulomb interaction, denoted by ${V}_{nk,m{k}^{{\prime} }}$ is replaced by the Morel-Anderson pseudopotential μ*, which is constant and has a value in the range of 0.1 − 0.2 depending on the material^69^. *N*_*F*_ is the electronic density of states at the Fermi Level. The anisotropic Eliashberg equations are solved at specific temperatures. The highest temperature yielding a non-trivial gap (e.g., Δ(*n***k**, *iω*_*l*_) ≠ 0) is the critical temperature *T*_C_^69^. The anisotropic electron-phonon coupling strength $\lambda (n{{{\bf{k}}}},m{{{{\bf{k}}}}}^{{\prime} },l-{l}^{{\prime} })$ is given by

8 $$\lambda (n{{{\bf{k}}}},m{{{{\bf{k}}}}}^{{\prime} },l-{l}^{{\prime} })=\int _{0}^{\infty }d\omega \,\frac{2\omega }{{({\omega }_{l}-{\omega }_{{l}^{{\prime} }})}^{2}+{\omega }^{2}}\,{\alpha }^{2}F(n{{{\bf{k}}}},m{{{{\bf{k}}}}}^{{\prime} },\omega ),$$

where the anisotropic Eliashberg spectral function ${\alpha }^{2}F(nk,m{k}^{{\prime} },\omega )$ is given by

9 $${\alpha }^{2}F(n{{{\bf{k}}}},m{{{{\bf{k}}}}}^{{\prime} },\omega )={N}_{F}{\sum }_{\nu }{\left|{g}_{mn,\nu }^{SE}({{{\bf{k}}}},{{{\bf{q}}}})\right|}^{2}\delta \,\left(\omega -{\omega }_{\nu },\,{{{\bf{q}}}}={{{\bf{k}}}}-{{{{\bf{k}}}}}^{{\prime} }\right)$$

where ${g}_{mn,\nu }^{SE}({{{\bf{k}}}},{{{\bf{q}}}})=\sqrt{\frac{1}{2{\omega }_{\nu {{{\bf{q}}}}}}}\left\langle m,{{{\bf{k}}}}+{{{\bf{q}}}}\left|{\delta }_{\nu {{{\bf{q}}}}}V\right|n,{{{\bf{k}}}}\right\rangle$ is the screened electron–phonon matrix element with initial electronic state $\left|n,{{{\bf{k}}}}\right\rangle$ and final state $\left|m,{{{\bf{k}}}}+{{{\bf{q}}}}\right\rangle$ and the change of the ion-electron potential *δ*_*ν***q**_*V* induced by phonons from branch *ν* with momentum **q**.

Averaging over **k** and ${{{{\bf{k}}}}}^{{\prime} }$ yields an isotropic Eliashberg spectral function *α*^2^*F*(*ω*) given by

10 $${\alpha }^{2}F(\omega )={\sum }_{n{{{\bf{k}}}},m{{{{\bf{k}}}}}^{{\prime} }}{W}_{n{{{\bf{k}}}}}{W}_{m{{{{\bf{k}}}}}^{{\prime} }}{\alpha }^{2}F(n{{{\bf{k}}}},m{{{{\bf{k}}}}}^{{\prime} },\omega ),$$

and can be interpreted as phonon density of states, where the density is weighted by the screened electron–phonon matrix element ${g}_{mn,\nu }^{SE}({{{\bf{k}}}},{{{\bf{q}}}})$. *W*_*n***k**_ = *δ*(*ϵ*_*n***k**_)/*N*_*F*_, with the density of states at the Fermi energy *N*_*F*_.

The cumulative electron–phonon coupling strength is given by

11 $$\lambda (\omega )=2\int _{0}^{\omega }d{\omega }^{{\prime} }\frac{{\alpha }^{2}F({\omega }^{{\prime} })}{{\omega }^{{\prime} }},$$

The total electron-phonon coupling strength *λ* is given by setting the upper bound of the integral *ω* beyond the highest phonon frequency.

The computation of the Eliashberg spectral function *α*^2^*F*(*ω*) and the solution of the Eliashberg equations are carried out using the EPW code (Electron–Phonon coupling using Wannier functions)^39,48^, which is implemented as a module within QE. EPW enables accurate interpolation of electron–phonon matrix elements from coarse Brillouin-zone grids, allowing efficient and converged calculations of superconducting properties and related quantities.

The interpolation is based on maximally localized Wannier functions (MLWFs)^49^, which provide a compact real-space representation of Bloch wave functions. These MLWFs are generated using the wannier90 code^70^. In practice, standard QE calculations are first performed to obtain the electronic structure and phonon properties on coarse **k**- and **q**-point meshes. The resulting quantities are then transformed into the Wannier representation, enabling EPW to interpolate them to much denser meshes that are required for the accurate evaluation of the Eliashberg spectral function and the numerical solution of the Eliashberg equations.

### Electron localization function

The electron localization function (ELF) is a quantitative measure of the degree of localization of electrons in atoms, molecules, or solids^45,46^. The concept is based on the probability of finding an electron in the vicinity of another reference electron with the same spin. Given a reference electron of spin *σ* at position **r**, the conditional probability of finding another electron with the same spin at a short distance *s* can be expanded to second order as

12 $${C}_{\sigma }({{{\bf{r}}}})={\sum }_{i=1}^{{N}_{\sigma }}{\left|\nabla {\varphi }_{i\sigma }({{{\bf{r}}}})\right|}^{2}-\frac{1}{4}\frac{{\left(\nabla {\rho }_{\sigma }({{{\bf{r}}}})\right)}^{2}}{{\rho }_{\sigma }({{{\bf{r}}}})},$$

where *φ*_*iσ*_(**r**) are the Kohn–Sham (KS) orbitals and *n*_*σ*_(**r**) is the spin-resolved electron density. Using the corresponding expression for a homogeneous free-electron gas (HFEG) as a reference, ${C}_{\sigma }^{{{{\rm{HFEG}}}}}({{{\bf{r}}}})=\frac{3}{5}{\left(6{\pi }^{2}\right)}^{2/3}{\rho }_{\sigma }{({{{\bf{r}}}})}^{5/3}$, the ELF is defined as

13 $${\eta }_{\sigma }({{{\bf{r}}}})={\left[1+{\left(\frac{{C}_{\sigma }({{{\bf{r}}}})}{{C}_{\sigma }^{{{{\rm{HFEG}}}}}({{{\bf{r}}}})}\right)}^{2}\right]}^{-1}.$$

The ELF takes values between 0 and 1. A value close to 1 indicates strong electron localization (as in covalent bonds or lone pairs), a value near 0 indicates complete delocalization, and *η* ≈ 0.5 corresponds to the reference localization of a homogeneous free-electron gas.

In QE, the ELF can be computed after a self-consistent field (SCF) calculation using the pp.x post-processing utility^67,68^. The KS orbitals and charge densities obtained from the SCF step are used to evaluate *C*_*σ*_(**r**) on a real-space grid, from which the ELF is calculated and written in formats suitable for visualization (e.g., .cube).

In this work, we do not consider magnetic materials; for that reason, we can omit the spin *σ* in our calculations.

### Computational details

All calculations were performed using Quantum ESPRESSO^67,68^ and the EPW code^39,48^ for the evaluation of the electronic structure, phonon dispersions, electron–phonon coupling, and superconducting properties. For MgB_2_ and Pb, the electronic structure was obtained using the Local Density Approximation (LDA) in the Perdew–Zunger (PZ) parameterization^71^, together with norm-conserving pseudopotentials, while for Nb we employed the Generalized Gradient Approximation (GGA) with the Perdew–Burke–Ernzerhof (PBE) functional^72^, also with norm-conserving pseudopotentials. The kinetic energy cutoffs were set to 60 Ry, 80 Ry, and 100 Ry for MgB_2_, Pb, and Nb, respectively. A Monkhorst–Pack **k**-grid^73^ centered at *Γ* was used with sizes 24 × 24 × 24 for MgB_2_, 16 × 16 × 16 for Pb, and 18 × 18 × 18 for Nb. For Brillouin-zone integrations, Marzari–Vanderbilt smearing^73^ with a width of 0.02 Ry was used for MgB_2_, Methfessel–Paxton smearing^74^ with a width of 0.025 Ry for Pb, and Marzari–Vanderbilt smearing with a width of 0.05 Ry for Nb. Phonon calculations were performed on coarse **q**-grids of 6 × 6 × 6 for MgB_2_ and 8 × 8 × 8 for Pb and Nb, which were also used for the construction of maximally localized Wannier functions. For MgB_2_, the initial projections were the *p*_*z*_ orbital for each B atom and three *s* orbitals located at fractional coordinates (0, 1.0, 0.5), (0.0, 0.5, 0.5), and (0.5, 0.5, 0.5) in crystal coordinates. For Pb, the initial projections corresponded to *sp*^3^ hybrid orbitals on each Pb atom, and for Nb, *d* orbitals on each Nb atom were used. Electron–phonon couplings in the Bloch basis were computed on the coarse **k**- and **q**-grids and then interpolated to fine **k**- and **q**-grids of 60 × 60 × 60 and 30 × 30 × 30 for MgB_2_, and 40 × 40 × 40 for both **k**- and **q**-grids in Pb and Nb. The Coulomb pseudopotential μ* was set to 0.16 for MgB_2_ and Nb, and 0.10 for Pb. The Matsubara frequency cutoffs were 1 eV for MgB_2_ and 0.1 eV for Pb and Nb. Dirac broadenings for electrons were fixed to 0.1 eV in all cases, while phonon broadenings were 0.05 meV for MgB_2_ and 0.5 meV for Pb and Nb. Inside the cavity, the same computational parameters were employed, with the addition of the electron–photon exchange potential within LDA when solving the Kohn–Sham equations. A photon energy of 70 meV was chosen for all materials, and the dimensionless ratio of the mode strength to the photon frequency, *λ*_*α*_/*ω*_*α*_, was varied from 0.14 to 1.4. For multiple photon modes (e.g., in-plane polarization), the electron–photon exchange potential was weighted accordingly (e.g., by a factor of 1/2).

## Data Availability

The data that support the findings of this study are available from the corresponding author upon request.

## Code availability

The codes associated with this manuscript are available from the corresponding author upon request.
